# Clinical Response and Improvement of the Quality of Life, Fatigue, Mood, and Sleep Disturbances During Vedolizumab Induction Therapy in Polish Patients With Moderate‐to‐Severe Ulcerative Colitis or Crohn’s Disease

**DOI:** 10.1155/cjgh/9955621

**Published:** 2026-06-02

**Authors:** Agata Michalak, Iwona Zawada, Anna Deskur, Maria Kopertowska-Majchrzak, Renata Talar-Wojnarowska, Danuta Domżał-Magrowska, Piotr Eder, Kamila Stawczyk-Eder, Katarzyna Waszak, Grażyna Rydzewska-Wyszkowska, Katarzyna Maciejewska, Katarzyna Karłowicz, Anita Gąsiorowska, Aleksandra Kaczka, Łukasz Konecki, Michał Krogulecki, Aleksandra Filipiuk, Maria Kłopocka, Ariel Liebert, Marek Hartleb, Magdalena Gawron-Kiszka, Elżbieta Poniewierka, Iga Gromny, Edyta Zagórowicz, Rafał Filip, Eliza Majewska, Halina Cichoż-Lach

**Affiliations:** ^1^ Department of Gastroenterology With Endoscopic Unit, Medical University of Lublin, Lublin, Poland, umlub.pl; ^2^ Department of Gastroenterology, Prof. Tadeusz Sokołowski’s University Clinical Hospital No. 1 of the Pomeranian Medical University, Szczecin, Poland; ^3^ Department of Internal Diseases, General Hospital, Międzychód, Poland, ghlagos.org.ng; ^4^ Department of Digestive Tract Diseases, Medical University of Lodz, Lodz, Poland, umed.pl; ^5^ Department of Gastroenterology, Dietetics and Internal Medicine, Poznan University Clinical Hospital, Poznan University of Medical Sciences, Poznan, Poland, ump.edu.pl; ^6^ Department of Gastroenterology and Internal Medicine, National Medical Institute of the Ministry of Interior and Administration, Warsaw, Poland; ^7^ Department of Gastroenterology, Medical University of Lodz, Lodz, Poland, umed.pl; ^8^ Department of Gastroenterology, Military Institute of Medicine, Warsaw, Poland, wim.mil.pl; ^9^ Department of Gastroenterology and Nutritional Disorders, Collegium Medicum in Bydgoszcz, Nicolaus Copernicus University in Torun, Bydgoszcz, Poland, umk.pl; ^10^ Department of Gastroenterology and Hepatology, Medical University of Silesia, Katowice, Poland, sum.edu.pl; ^11^ Department of Gastroenterology and Hepatology, Wroclaw Medical University, Wroclaw, Poland, umed.wroc.pl; ^12^ Department of Gastroenterology, Hepatology and Internal Medicine, Wroclaw Medical University, Wroclaw, Poland, umed.wroc.pl; ^13^ Department of Oncological Gastroenterology, The Maria Sklodowska-Curie National Research Institute of Oncology, Warsaw, Poland, karolinska.se; ^14^ Department of Gastroenterology, Hepatology and Clinical Oncology, Centre of Postgraduate Medical Education, Warsaw, Poland; ^15^ Department of Gastroenterology of the Centre for Comprehensive Treatment of Inflammatory Bowel Diseases, Clinical Regional Hospital No. 2, Rzeszow, Poland; ^16^ Department of Medicine, Medical College of Rzeszow University, Rzeszow, Poland; ^17^ Medical Department, Takeda Pharma sp. z o.o, Warsaw, Poland

**Keywords:** Crohn’s disease, inflammatory bowel disease, quality of life, ulcerative colitis, vedolizumab

## Abstract

**Background:**

Inflammatory bowel disease (IBD) encompasses chronic gastrointestinal disorders that significantly impact patients’ quality of life (QoL). While clinical trials support the efficacy and safety of vedolizumab, real‐world data linking its effectiveness to QoL‐related patient‐reported outcomes remain limited. This prospective observational study assessed the effects of 14‐week vedolizumab induction therapy on QoL (i.e., fatigue, mood, and sleep disturbances), clinical response, safety, and predictors of response.

**Methods:**

A total of 257 patients with ulcerative colitis (UC; *n* = 205) and Crohn’s disease (CD; *n* = 52) received 14‐week vedolizumab induction therapy. Disease activity, QoL (assessed using the total Inflammatory Bowel Disease Questionnaire [IBDQ]), fatigue (Functional Assessment of Chronic Illness Therapy‐Fatigue [FACIT‐Fatigue]), mood and sleep disturbances (PROMIS Depression and Sleep Disturbance scales), and selected biomarkers were evaluated. Clinical response was assessed using the Mayo score for UC and Crohn’s Disease Activity Index (CDAI) score for CD.

**Results:**

At Week 14, clinical response was observed in 82% of UC patients and 82.7% of CD patients. Median total IBDQ scores increased by 45.0 points in UC and 31.5 points in CD (*p* < 0.001). Median prorated FACIT‐Fatigue scores increased by 5.0 points in both groups (*p* < 0.001), while median prorated PROMIS Sleep Disturbance and Depression T‐scores decreased (*p* < 0.05). Improvement in QoL was moderately correlated with clinical response (*r* = 0.5). In logistic regression analysis, baseline patient characteristics indicative of advanced disease were associated with a reduced likelihood of clinical and QoL response to treatment. A total of 28 adverse events were reported, including 11 serious events and four treatment‐related events.

**Conclusions:**

Vedolizumab reduced disease activity and led to a rapid improvement in QoL during the 14‐week induction therapy in patients with IBD. Clinical response was positively correlated with QoL improvement. Predictors of reduced response were consistent with features of advanced disease.

## 1. Introduction

Ulcerative colitis (UC) and Crohn’s disease (CD) are the most common types of inflammatory bowel disease (IBD), characterized by symptoms such as abdominal pain, diarrhea, weight loss, anemia, and fatigue [1–3]. In Europe, the prevalence of CD and UC ranges from 1.5 to 213 cases per 100,000 and from 2.4 to 294 per 100,000, respectively, affecting an estimated 0.2% of the population [4, 5]. In Poland, prevalence rates were 61.6 per 100,000 for CD and 191.6 per 100,000 for UC in 2020 [6].

Disease progression in IBD can lead to functional decline, disability, and structural bowel damage [4, 5]. Treatment options include corticosteroids, immunosuppressants, biologics, and small‐molecule therapies. According to the STRIDE II consensus, current treat‐to‐target strategies define clinical response as an immediate treatment goal, while endoscopic healing and normalization of health‐related quality of life (QoL) are long‐term goals [7].

UC and CD are lifelong conditions with alternating periods of relapse and remission that often contribute to poor QoL [2]. Symptoms of IBD impair social functioning and multiple aspects of life [8]. Studies have shown that patients with IBD experience higher rates of comorbid depression and anxiety compared to healthy controls [8–11]. Fatigue and sleep disturbances associated with IBD can exacerbate mental health symptoms and further diminish QoL [12, 13]. Several patient‐reported outcome (PRO) scores have been developed to evaluate QoL in IBD, including the Inflammatory Bowel Disease Questionnaire (IBDQ), which is widely used in clinical research [13]. The Functional Assessment of Chronic Illness Therapy‐Fatigue (FACIT‐Fatigue) and Patient‐Reported Outcomes Measurement Information System (PROMIS) scales have also been validated for assessing fatigue and self‐reported mental and physical health [14–17]. Despite the availability of these tools and the recognized burden of IBD on QoL, data linking QoL to clinical response rates and biomarkers remain limited [12].

Vedolizumab (Entyvio; Takeda Pharmaceuticals) is a gut‐selective antilymphocyte trafficking integrin monoclonal antibody approved for the treatment of moderately to severely active UC and CD [13, 18]. Its efficacy and safety have been demonstrated in several Phase 3 clinical trials [3, 19]. In Poland, vedolizumab is approved for both induction and maintenance therapy under the national drug program, and its use has been evaluated in prospective real‐world studies. Observational data from Polish UC and CD cohorts have confirmed the effectiveness and safety of vedolizumab for both induction and maintenance therapy, even in patients with high disease severity and delayed initiation of biologic treatment [3, 20].

The primary endpoint of this prospective observational study (KUJAWIAK) was to determine the effect of vedolizumab on patient‐reported QoL, measured as a change in the IBDQ score from baseline to Week 14. Secondary endpoints included clinical response to treatment at Week 14, changes in FACIT‐Fatigue, PROMIS Depression, and PROMIS Sleep Disturbance scores, and real‐world safety of vedolizumab. Exploratory objectives included examining correlations among biomarkers, disease activity, and QoL‐related PROs, as well as identifying predictors of treatment response.

## 2. Materials and Methods

### 2.1. Study Design and Participants

KUJAWIAK was a prospective, noninterventional, single‐arm, open‐label, multicenter study conducted between September 2020 and November 2022. The recruitment period lasted 19 months for UC patients and 23 months for CD patients. No predefined sample size was established; however, enrollment of up to 300 consecutive patients eligible for treatment under the national drug program was estimated. Of the 257 patients enrolled across 13 gastroenterology centers in Poland, 205 initiated vedolizumab induction therapy for UC and 52 for CD. The study protocol was approved by the Bioethics Committee of the Nicolaus Copernicus University in Toruń, Collegium Medicum in Bydgoszcz (Approval No. KB 36312020), and the study was registered as EUPAS34232. Written informed consent was obtained from all participants. Eligible patients were aged ≥ 18 years and met the criteria for vedolizumab treatment under the drug program between June 2020 and September 2022. Patients enrolled in any interventional clinical trial or deemed unfit for the study by the investigator were excluded.

Patients with UC were eligible for vedolizumab induction under the drug program if they had moderate‐to‐severe disease (defined as a total Mayo score of > 6), a contraindication to ciclosporin treatment, and inadequate response, intolerance, or contraindication to standard treatment [21]. Patients with CD were eligible if they had moderate‐to‐severe disease (defined as a Crohn’s Disease Activity Index [CDAI] score of > 300), along with inadequate response, intolerance, or contraindication to glucocorticoid or immunosuppressant treatment [21].

### 2.2. Study Assessments

Participants attended four visits at Weeks 0, 2, 6, and 14, during which they completed paper PRO questionnaires prior to receiving vedolizumab. Collected PROs comprised FACIT‐Fatigue, PROMIS Depression, and PROMIS Sleep Disturbance scores [13–15]. Additional data included demographic characteristics, clinical characteristics of UC or CD, biomarkers (such as C‐reactive protein [CRP], hemoglobin, hematocrit, red blood cells [RBCs], mean corpuscular volume [MCV], mean corpuscular hemoglobin [MCH], and platelet count), and concomitant medications. All assessments were conducted as part of routine clinical practice.

### 2.3. Study Endpoints

The primary endpoint was to assess changes in the IBDQ score from baseline to Week 14 and the proportion of patients who achieved a response to treatment. An IBDQ response was defined as an increase of at least 16 points, consistent with the minimal clinically important difference for this scale [22]. Secondary endpoints included clinical response at Week 14 (defined as a decrease in the Mayo score from baseline by at least 3 points for UC or a decrease in the CDAI score by at least 70 points and at least 25% from baseline for CD), as well as the effects of vedolizumab on fatigue (FACIT‐Fatigue scale), mood (PROMIS Depression), and sleep disturbances (PROMIS Sleep Disturbance). FACIT‐Fatigue scores below 30 were considered indicative of severe fatigue [16]. Real‐world safety outcomes were also evaluated. Exploratory objectives included assessing correlations between disease activity and QoL‐related PROs, identifying potential predictors of treatment response, and characterizing the study population.

### 2.4. Statistical Analyses

The full analysis set (FAS) comprised all enrolled participants, regardless of whether they received vedolizumab. The safety population consisted of patients who received at least one dose of vedolizumab, while the efficacy population included those who completed the study.

Continuous variables were presented as median with range (minimum–maximum) or mean with standard deviation, as appropriate. Categorical variables were reported as percentages. Endpoints were analyzed using 95% confidence intervals (CIs). Missing data were substituted using the last observation carried forward method. Group comparisons were made using the paired Student’s *t* test or the Wilcoxon signed‐rank test, depending on the assessment of data normality. Statistical significance was set at *p* value < 0.05.

Correlations were assessed between selected biomarkers (CRP, hematocrit, hemoglobin, MCH, MCV, platelet count, and RBC), clinical parameters, and PRO measures. Multivariate logistic regression models were used to identify independent baseline predictors of clinical or IBDQ response at Week 14. Model selection was based on the Akaike Information Criterion using a backward stepwise procedure. A two‐tailed *p* value < 0.05 was considered significant.

Incidence rates of adverse events (AEs) were calculated using person‐time analysis.

Statistical analyses were performed using R Version 4.0.3 (R Foundation for Statistical Computing, c/o Institute for Statistics and Mathematics Wirtschaftsuniversität, Vienna, Austria).

## 3. Results

### 3.1. Study Population and Treatment Exposure

**FIGURE 1:**
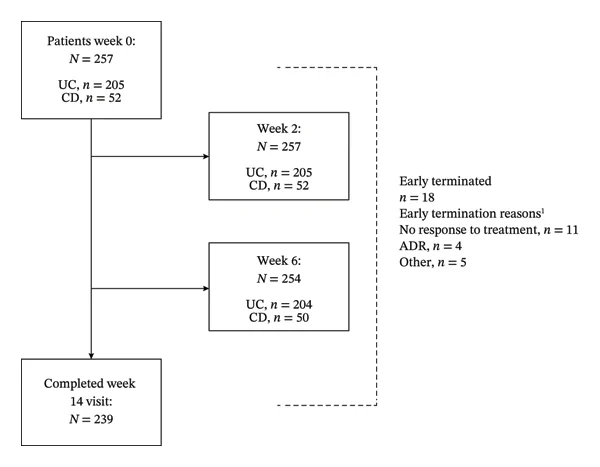
Patient disposition, including reasons for termination. 1. More than one reason for termination could be provided. Abbreviations: ADR, adverse drug reaction; CD, Crohn’s disease; UC, ulcerative colitis.

A total of 257 patients were enrolled in the study, including 205 patients with UC and 52 with CD (Figure 1). Early termination occurred in 18 patients (7.0%), comprising 13 patients with UC and 5 with CD. The primary reasons for discontinuation were lack of response to treatment (11 patients: seven with UC and four with CD) and adverse drug reaction (four patients: all with UC) (Table 1). At enrollment, patients with UC had a median Mayo score of 10 and a median disease duration of 7 years (range: 0–47), while those with CD had a median CDAI score of 328 and a median disease duration of 8.5 years (range: 1–29). Previous exposure to biologic treatment, defined as receiving at least one dose of a biologic agent before enrollment, was reported in 94 UC patients (45.9%) and 30 CD patients (57.7%). At baseline, 24 patients (9.4%) were using psychoanaleptic or psycholeptic medication (such as benzodiazepine derivatives or selective serotonin reuptake inhibitors) as concomitant medication. Additional patient characteristics and treatment data are presented in Table 2 and Supporting Tables 1 and 1. Patients received treatment in accordance with the local Entyvio label. The median number of vedolizumab doses was 4.0 for both UC and CD patients. An additional infusion at Week 10 was administered to 10 patients with CD (21.3%). All patients received the initial induction dose at Week 0, and the first maintenance dose at Week 14 was administered to 87.5% of UC patients and 91.5% of CD patients.

**TABLE 1:** Classification of adverse events (FAS population).

Safety information, *n* (%)	Overall, *N* = 257 (%)	Patients with UC, *n* = 205 (%)	Patients with CD, *n* = 52 (%)
Serious infections^1^	6 (2.3)	5 (2.4)	1 (1.9)
Other clinically significant infections	7 (2.7)	5 (2.4)	2 (3.8)
Infusion‐related reactions	1 (0.4)	1 (0.5)	0 (0.0)
All other AEs, SAEs, and adverse reactions	13 (5.1)	10 (4.9)	3 (5.8
Lack of effect	1 (0.4)	0 (0.0)	1 (1.9)

^1^atypical pneumonia in one patient, upper respiratory tract infection in one patient, *Clostridium difficile* infection in two patients, *Clostridium difficile* colitis in two patients.

Abbreviations: AE, adverse event; CD, Crohn’s disease; FAS, full analysis set; SAE, serious adverse event; UC, ulcerative colitis.

**TABLE 2:** Baseline patient characteristics (FAS population)**.**

Parameter	Overall, *N* = 257	Patients with UC, *n* = 205	Patients with CD, *n* = 52
Mayo score, median (min–max)	NA	10.0 (7.0–12.0)	NA
CDAI score, median (min–max)	NA	NA	328.0 (302.0–488.0)
Age, median (min–max), years	34.0 (18.0–74.0)	35.0 (18.0–74.0)	34.0 (19.0–67.0)
Female sex, *n* (%)	100 (38.9%)	75 (36.6%)	25 (48.1%)
Disease duration, median (min–max), years	7.0 (0.0–47.0)	7.0 (0.0–47.0)	8.5 (1.0–29.0)
Extraintestinal symptoms, *n* (%)	81 (31.5%)	53 (25.9%)	28 (53.8%)
Oral corticosteroids (prior)^1^, *n* (%)	249 (96.9%)	201 (98.0%)	48 (92.3%)
Oral corticosteroids (baseline)^2^, *n* (%)	177 (68.9%)	149 (73.7%)	28 (53.8%)
Immunomodulatory drugs^1^, *n* (%)	230 (89.5%)	184 (89.8%)	46 (88.5%)
Previous biologic treatment, *n* (%)	124 (48.2%)	94 (45.9%)	30 (57.7)
Naïve to previous biologic treatment, *n* (%)	133 (51.8%)	111 (54.1%)	22 (42.7%)
Failed biologic treatment, *n* (%)	62 (24.1%)	45 (22%)	17 (32.7%)
CRP^3^, mg/L, median (min‐max)	5.0 (0.1–207.0)	4.5 (0.1–207.0)	9.0 (0.2–114.0)

Abbreviations: CD, Crohn’s disease; CDAI, Crohn’s Disease Activity Index; CRP, C‐reactive protein; FAS, full analysis set; UC, ulcerative colitis.

^1^treatment in patients’ prior medical history.

^2^current corticosteroid use at baseline.

^3^missing data *n* = 1.

### 3.2. Clinical Response to Treatment

In UC patients, the Mayo score decreased by a median of 6.0 points from baseline to Week 14 (range: 11.0–3.0; *p* < 0.001), with 82.0% achieving a clinical response. Among patients with CD, the CDAI score decreased by a median of 198.5 points (range: 371.0–84.0; *p* < 0.001), with 82.7% of patients demonstrating a clinical response to treatment.

### 3.3. PROs

A significant improvement in QoL was observed in both UC and CD patients from baseline to Week 14, as measured by the median IBDQ score (UC: 125.0 to 170.0 points, *p* < 0.001; CD: 132.0 to 163.5 points, *p* < 0.001). IBDQ scores increased at Weeks 2, 6, and 14 in both groups (*p* < 0.001), indicating overall QoL improvement (Figure 2 and Supporting Table 1). Clinically meaningful QoL improvement from baseline to Week 14 was achieved in 62.9% of UC patients (95% CI: 55.9%–69.5%) and 57.7% of CD patients (95% CI: 43.3%–71.0%). This improvement was significant across all patient subgroups, regardless of prior exposure to biologic treatment.

FIGURE 2Change from baseline in patient‐reported outcome scores at Weeks 2, 6, and 14. Median changes from baseline (Week 0) are presented for IBDQ, FACIT‐Fatigue (prorated scores), PROMIS Depression (prorated T‐scores), and PROMIS Sleep Disturbance (prorated T‐scores) in (a) UC and (b) CD patients. Numbers above bars indicate mean change values, and whiskers represent the limits of 95% confidence intervals; ^∗∗∗^ *p* < 0.001; ^∗∗^0.001 ≤ *p* < 0.01; ^∗^0.01 ≤ *p* < 0.05. Statistical analysis was conducted using the paired *t*‐test or Wilcoxon signed‐rank test. Abbreviations: CD, Crohn’s disease; FACIT‐Fatigue, Functional Assessment of Chronic Illness Therapy‐Fatigue; IBDQ, Inflammatory Bowel Disease Questionnaire; PROMIS, Patient‐Reported Outcomes Measurement Information System; vs., versus; UC, ulcerative colitis.

**Figure:**
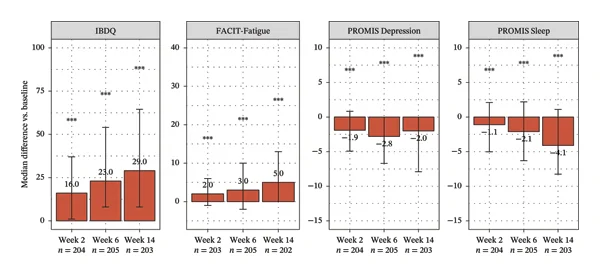
(a)

**Figure:**
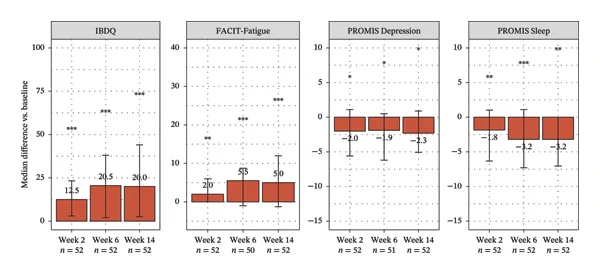
(b)

The median FACIT‐Fatigue score increased by 5.0 points from baseline to Week 14 in both groups (range: −27.0–39.0, *p* < 0.001, for UC; range: −11.0–35.0, *p* < 0.001, for CD). A significant increase was observed between Weeks 2, 6, and 14, and this effect was maintained in both biologic‐experienced and biologic‐naïve patients (Figure 2 and Supporting Table 1).

In patients with UC, the median prorated PROMIS T‐score decreased by 4.1 points for sleep disturbances (range: −40.1–31.5, *p* < 0.001) and by 2.0 points for depression (range: −34.3–20.0, *p* < 0.001) from baseline to Week 14. In patients with CD, the median change was −3.2 points for sleep disturbances (range: −31.6–10.9, *p* = 0.001) and −2.3 points for depression (range: −32.9–14.6, *p* = 0.038). Median prorated T‐scores for both domains declined steadily over the course of treatment (Figure 2 and Supporting Table 1).

Changes in PRO scores in patients without clinical response to treatment were numerically smaller than in responders, achieving statistical significance relative to baseline only in the latter subgroup. This difference was especially pronounced in UC patients, where the comparison between PRO response levels in responders and nonresponders revealed significant differences (*p* < 0.05; Supporting Table 1).

During the study, 11.7% of patients (*n* = 30) reported using psychoactive medication at any time. A comparison of changes in PRO response levels between these patients and those who did not use psychoactive medication showed no significant differences in the magnitude of change from baseline to Week 14 (*p* > 0.05; Supporting Table 1).

### 3.4. Biomarkers

In UC patients, median CRP levels decreased from 4.5 mg/L (range: 0.1–207) at baseline to 3.1 mg/L (range: 0.1–72.3) at Week 14 (*p* = 0.005). Additionally, median levels of hemoglobin, hematocrit, RBC, and MCV significantly increased at Week 14 compared to baseline (by 0.5 g/dL, 1.1%, 0.1 × 10^12^/L, and −0.4 fL, respectively). In CD patients, we observed numerical improvement in CRP, hemoglobin, and hematocrit; however, the differences were not statistically significant (Supporting Table 1). CRP levels decreased significantly in UC patients with prior exposure to biologic treatment, from a median of 5.7 mg/L (range: 0.1–73.4) to 4.2 mg/L (range: 0.3–32.8, *p* = 0.003), and in patients who failed previous biologic treatment, from a median of 7.5 mg/L (range: 0.1–64.6) to 4.7 mg/L (range: 0.3–22.9, *p* = 0.004).

### 3.5. Associations Between Outcomes

In linear regression analyses, the Mayo score demonstrated a moderate inverse correlation with the IBDQ total score (*r* = −0.52, *p* < 0.001) and a moderate positive correlation with the PROMIS prorated T‐score for depression (*r* = 0.35, *p* < 0.001) (Figure 3(a)). Similarly, the CDAI score showed a moderate inverse correlation with IBDQ total scores (*r* = −0.5, *p* < 0.001) and a moderate positive correlation with the PROMIS Depression score (*r* = 0.41, *p* = 0.003) (Figure 3(b)). Strong correlations were observed between FACIT‐Fatigue and IBDQ scores in both UC (*r* = 0.75, *p* < 0.001) and CD patients (*r* = 0.72, *p* < 0.001), and between hematocrit and hemoglobin levels (*r* = 0.94, *p* < 0.001). Moderate correlations were also noted between PROMIS scores and other PRO measures in both UC (*p* < 0.001) and CD patients (*p* ≤ 0.006).

FIGURE 3Sensitivity analysis heatmaps for differences in Mayo/CDAI, IBDQ, FACIT‐Fatigue, PROMIS scores, CRP, hemoglobin, and hematocrit levels in (a) UC and (b) CD patients. Crossed values indicate correlations that are not statistically significant. Abbreviations: CD, Crohn’s disease; CDAI, Crohn’s Disease Activity Index; CRP, C‐reactive protein; Depr, depression; FACIT, Functional Assessment of Chronic Illness Therapy‐Fatigue; IBDQ, Inflammatory Bowel Disease Questionnaire; PROMIS, Patient‐Reported Outcomes Measurement Information System; vs., versus; UC, ulcerative colitis.

**Figure:**
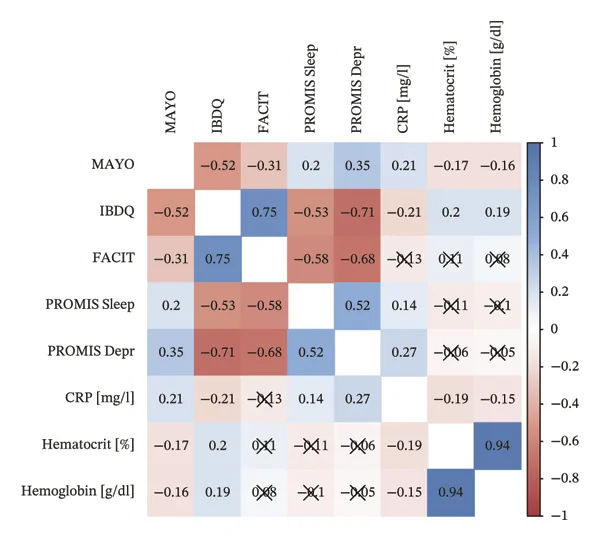
(a)

**Figure:**
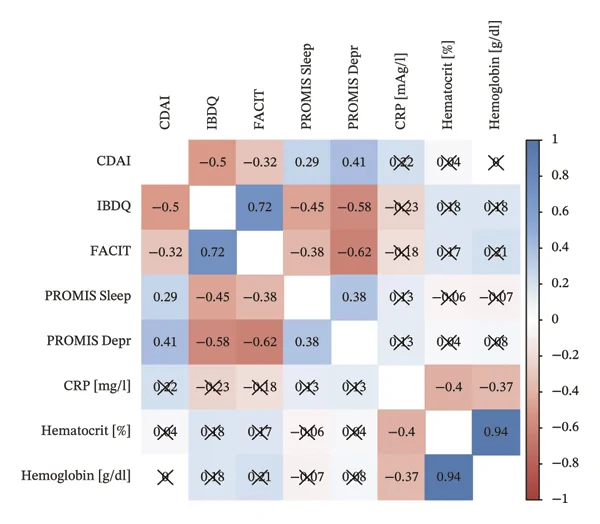
(b)

### 3.6. Sensitivity Analysis

#### 3.6.1. Predictors of Response in UC

In a multivariate logistic regression analysis of predictors of clinical response in patients with UC, baseline characteristics were evaluated. Severe rectal bleeding at baseline, corresponding to a score of 3 on the Mayo Rectal Bleeding Subscale, was identified as a significant negative predictor of clinical response (odds ratio [OR]: 0.27; 95% CI: 0.09–0.82, *p* = 0.017) (Table 3). In a multivariate analysis of predictors of QoL response, male sex (OR: 0.346, 95% CI: 0.175–0.657, *p* = 0.002), older age (OR: 0.974, 95% CI: 0.951–0.997, *p* = 0.029), and severe rectal bleeding (OR: 0.315, 95% CI: 0.096–0.965, *p* = 0.047) were identified as significant negative predictors (Table 3).

**TABLE 3:** Logistic regression model to predict clinical or IBDQ response at week 14 in UC patients (FAS).

Variable	OR	95% CI	*p* value
*A*			
Rectal bleeding category: severe^1^	0.27	0.09–0.82	0.017
Immunomodulators: yes			

*B*			
Sex: male	0.346	0.175–0.657	0.002
Age (years)	0.974	0.951–0.997	0.029
Rectal bleeding category: severe^1^	0.315	0.096–0.965	0.047

*Note:* To describe the relation between UC patients’ demographic and clinical characteristics, multivariate logistic regression models were created, explaining clinical response (A) and IBDQ response (B) scores using disease activity (assessed by the Mayo score) and adjusted for baseline score, sex, age, body mass index, smoking status, and clinical characteristics of the disease.

Abbreviations: CI, confidence interval; FAS, full analysis set; IBDQ, Inflammatory Bowel Disease Questionnaire; OR, odds ratio.

^1^Score 3 in the Rectal Bleeding Scale of the Mayo score.

#### 3.6.2. Predictors of Response in CD

In a multivariate logistic regression analysis of patients with CD, baseline predictors of clinical response were evaluated. Significant predictors of achieving a clinical response included Montreal classification localization L3 (OR: 13.396, 95% CI: 1.293–138.765, *p* = 0.030), time from diagnosis > 5 years (OR: 10.578, 95% CI: 1.067–104.896, *p* = 0.044), and hospitalization due to CD exacerbation in the previous year (OR: 11.001, 95% CI: 1.363–88.820, *p* = 0.024). Although prior exposure to biologic treatment was not a significant predictor (OR: 0.105, 95% CI: 0.008–1.390, *p* = 0.087), it contributed to an improved model fit. Previous corticosteroid use (OR: 0.117, 95% CI: 0.014–0.974, *p* = 0.047) was associated with a lower likelihood of clinical response (Table 4). In a multivariate analysis of predictors of IBDQ response in patients with CD, a higher abdominal pain score (OR: 0.847, 95% CI: 0.728–0.984, *p* = 0.030) and prior exposure to biologic treatment (OR: 0.116, 95% CI: 0.025–0.547, *p* = 0.006) were negative predictors of achieving a clinically meaningful improvement in QoL. In contrast, the ileocolonic disease location was a positive predictor (OR: 4.554, 95% CI: 1.026–20.204, *p* = 0.046) (Table 4).

**TABLE 4:** Logistic regression model to predict clinical or IBDQ response at week 14 in CD patients (FAS).

Variable	OR	95% CI	*p* value
*A*			
Previous biologic treatment: yes	0.105	0.008–1.390	0.087
Failed biologic treatment: yes			
Time from diagnosis: more than 5 years	10.578	1.067–104.896	0.044
Localization: L3	13.396	1.293–138.765	0.030
Hospitalization because of worsening of CD in the past year: yes	11.001	1.363–88.820	0.024
Corticosteroids: yes	0.117	0.014–0.974	0.047

*B*			
Abdominal pain score	0.847	0.728–0.984	0.030
CDAI total score	1.011	0.997–1.025	0.138
Previous biologic treatment: yes	0.116	0.025–0.547	0.006
Localization: L3	4.554	1.026–20.204	0.046

*Note:* To describe the relation between CD patients’ demographic and clinical characteristics, multivariate logistic regression models were created, explaining clinical response (A) and IBDQ response (B) scores using disease activity (assessed by the CDAI score) and adjusted for baseline score, sex, age, body mass index, smoking status, and clinical characteristics of the disease.

Abbreviations: CDAI, Crohn’s Disease Activity Index; CI, confidence interval; FAS, full analysis set; IBDQ, Inflammatory Bowel Disease Questionnaire; OR, odds ratio.

### 3.7. Safety

Throughout the study, 28 AEs were reported in 26 patients (10.1%), including 20 with UC and 8 with CD. Serious AEs occurred in 11 patients, and 4 patients (1.6%) experienced treatment‐related AEs, including vomiting, hypersensitivity reaction, arthralgia, and psoriasis. The overall incidence rate of AEs was 35.8 per 100 patient‐years, with a rate of 17.2 per 100 patient‐years for serious AEs. A detailed classification of AEs is presented in Table 1.

## 4. Discussion

This study evaluated the impact of 14‐week vedolizumab induction therapy on QoL and clinical response in Polish patients with moderate‐to‐severe IBD. In addition, associations between study endpoints were examined, and baseline characteristics were assessed as potential predictors of clinical and QoL responses to treatment. To our knowledge, this is the first study to investigate QoL‐related PROs and predictors of response to vedolizumab induction in an Eastern European IBD cohort. Key findings include (1) high clinical response rates in UC and CD patients; (2) baseline characteristics indicative of low QoL, poor sleep quality, and high fatigue and depression levels; (3) rapid QoL improvement after 2 weeks of treatment; and (4) few predictors of response, generally consistent with disease severity.

In our study, clinical response rates at Week 14 reached 82% in patients with UC and 82.7% in those with CD, which is consistent with 14‐week response rates reported in Polish UC and CD cohorts and exceeding those observed in pivotal vedolizumab trials [2, 4, 20]. Real‐world studies reported response rates ranging from 35% to 61%, likely reflecting differences in response definitions, baseline disease severity, and prior treatments [23]. The median disease duration was 7.0 years for UC and 8.5 years for CD, with corresponding baseline median Mayo and CDAI scores of 10.0 and 326.0, respectively. Additionally, 45.9% of UC patients and 57.7% of CD patients had prior exposure to biologic treatment, which is consistent with previously published Polish data [2, 20]. In contrast, the GEMINI trials reported shorter median disease durations (4.6 for UC and 7.7 years for CD) and slightly lower baseline Mayo and CDAI scores (9.0 and 318.0, respectively) [24].

Impaired QoL is a major contributor to the overall disease burden in IBD. The STRIDE II initiative identifies endoscopic remission and restoration of QoL as essential long‐term treatment targets [25]. In the KUJAWIAK study, clinically meaningful QoL improvement was achieved by 62.9% of patients with UC and 57.5% of those with CD, aligning with results from the GEMINI, VARSITY, and VISIBLE1 trials despite differences in the study design [13, 19, 25, 26]. Smaller real‐world studies have also demonstrated QoL improvements during both induction and maintenance therapy [18, 20]. In our cohort, significant improvements in QoL were observed as early as 2 weeks after treatment initiation, with clinically meaningful levels reached at Week 2 in UC patients and at Week 6 in CD patients. These findings are consistent with post hoc analyses from the GEMINI trials, particularly among patients naïve to antitumor necrosis factor treatment [24]. However, data on the timing of QoL response during induction treatment remain limited. Notably, the GEMINI studies reported lower clinical response rates to 6‐week induction in CD compared to UC patients [13, 27]. Given the moderate to strong inverse correlations observed between clinical disease activity and IBDQ scores, the slower clinical response in CD may have accounted for the delayed achievement of minimal clinically important QoL improvement in CD patients in our study [20, 27].

Fatigue is a common and burdensome symptom in IBD, particularly during active disease [10]. Clinically meaningful improvements are estimated at 4–9 points for UC and 7–10 points for CD [16, 28]. In our study, median baseline FACIT‐Fatigue scores were 28.0 for UC and 26.0 for CD, reflecting severe fatigue in both groups. A median 5‐point improvement observed over 14 weeks of vedolizumab induction therapy was clinically meaningful in UC patients and aligns with findings from a long‐term Belgian IBD registry [29]. Notably, this study is among the first to report early improvements in fatigue during IBD treatment.

Sleep disturbances are reported by over 80% of IBD patients, compared to approximately 32% in the general population. Anxiety and depression affect 35% and 25% of individuals with IBD, respectively [30]. In our cohort, significant improvements were observed in PROMIS Depression and Sleep Disturbance scores during vedolizumab induction. However, clinically meaningful thresholds for these scales have not yet been established specifically for Polish patients. Nevertheless, our findings are consistent with previous studies reporting improvements in psychosocial outcomes during vedolizumab induction therapy [17].

Statistically significant improvements in PRO scores were only observed in IBD patients with clinical response to treatment. Consistently, clinically meaningful improvement of IBDQ scores was only reached in responders. PRO values differed significantly between responders and nonresponders in the UC group, but not in the CD group. The absence of significant differences in CD likely reflects limited statistical power due to the very small number of CD nonresponders (*n* = 9), rather than a fundamentally different response pattern. Overall, these findings underscore that symptomatic and QoL improvements during vedolizumab induction are closely linked to clinical response, consistent with recent findings from the UK‐wide IBD‐RESPONSE study [31].

Notably, a small proportion of patients in our study reported using psycholeptic and psychoanaleptic medications during the study, with no significant impact on changes in PRO scores at the end of treatment.

Improvements in biomarker levels observed in our study, particularly in CRP and hemoglobin, were consistent with previous research [2]. While these improvements were significant in the UC cohort, no significance was observed in the CD cohort, possibly due to the smaller size of this subgroup. It should be noted that fecal calprotectin levels were not routinely collected in IBD patients in Poland at the time this study was conducted, which limited our ability to analyze inflammatory biomarkers.

Our study revealed correlations between clinical disease activity and QoL, fatigue, mood, and sleep disturbances, which aligns with previous research linking these symptoms to active IBD and disease severity [30]. These relationships are likely bidirectional. The variability across published results may be partly attributable to the lack of standardized, universally accepted PRO scores in IBD.

In our study, UC and CD demonstrated distinct predictor profiles; however, the overall findings remained consistent across both diseases, suggesting that these differences might reflect underlying heterogeneity in disease characteristics, pathophysiology, and the imbalance in cohort sizes. Severe rectal bleeding at baseline was associated with a reduced likelihood of clinical response in UC at Week 14. This finding is consistent with previous reports linking greater disease severity (Mayo score ≥ 9) to diminished treatment response [32]. The observation that older age and male sex were associated with a lower likelihood of QoL improvement in UC patients remains unexplained and warrants further investigation. Among patients with CD, prior corticosteroid use and previous exposure to biologic treatment were associated with a lower probability of achieving a clinical response. Similarly, higher baseline abdominal pain scores and prior biologic exposure predicted a reduced likelihood of QoL improvement, which is consistent with previously published data [32].

Interestingly, in our cohort, hospitalization within the year before enrollment and longer disease duration (> 5 years) were associated with higher clinical response rates in CD at Week 14. Earlier literature has linked prior hospitalization to a higher risk of rehospitalization within 3 months [33], whereas our endpoint captured short‐term clinical response rather than healthcare utilization. This distinction in outcomes may explain the apparent discrepancy. In addition, among patients with severe disease, postdischarge transitional care components (e.g., early gastroenterology follow‐up and remote monitoring) could plausibly support short‐term symptom improvement and documentation of response, although IBD‐specific evidence for standardized discharge protocols affecting long‐term disease activity remains limited. Regarding disease duration, some reports suggest that earlier initiation of treatment (e.g., within ∼18–24 months of diagnosis) is associated with higher response rates, whereas longer durations are generally linked to lower responses [34]. However, when alternative cut‐offs such as 3 or 5 years are applied, no single time point across the disease continuum appears to consistently modify the treatment effect. The higher odds of clinical response in CD patients with a disease duration of more than 5 years in our study may reflect survivorship and care optimization phenomena (e.g., enrichment for disease phenotypes more amenable to gut‐selective therapy and more frequent follow‐up), which could influence observed responder rates in real‐world settings.

In our cohort, 61.5% of CD patients had ileocolonic (L3) disease location at baseline, which was associated with a higher likelihood of clinical and QoL improvement compared to previously published data [35]. However, the significance of disease location as a predictive factor should be interpreted with caution due to the exploratory nature of this analysis. Although some heterogeneity was observed between the UC and CD cohorts, our results overall suggest a reduced probability of achieving clinical and QoL response in patients with more severe disease and a history of extensive treatment. This has important implications for treatment strategies aimed at optimizing outcomes in the Polish IBD population, particularly given the limited access to biologic treatment.

Our findings are consistent with the established safety profile of vedolizumab. In the open‐label GEMINI LTS study, AE rates were reported at 122.1 per 100 patient‐years for UC patients and 179.9 per 100 patient‐years for CD patients, with serious AE rates of 9.1 and 14.7 per 100 patient‐years, respectively. The most frequently reported AE was disease exacerbation, occurring in 35.9% of patients with UC and 35.5% of those with CD. Infections were also common, affecting 66.1% of UC patients and 69.5% of those with CD [4].

We acknowledge that outcomes based on patient‐reported measures and unblinded clinical assessments are susceptible to expectation, reporting, and observer bias; therefore, effect sizes may be partly overestimated. In a meta‐analysis of randomized trials in UC, placebo and expectation effects on symptom‐based endpoints accounted for up to 33% of response and remission rates. Placebo effects were lower in multicenter studies compared to single‐site studies, and the longer disease duration (> 5 years) was associated with a lower placebo effect [36]. A similar magnitude of placebo effect has been reported in CD, with a longer disease duration and higher disease severity associated with lower susceptibility to bias [37]. In our study, we relied on established, validated tools and clinically meaningful thresholds wherever possible. Given the magnitude of clinical and PRO responses in a moderate‐to‐severe patient cohort, the alignment across validated clinical, patient‐reported, and biological outcomes supports the credibility and interpretability of our findings.

Additional limitations and potential sources of bias in this study include the exclusive enrollment of patients with moderate‐to‐severe disease, a relatively short follow‐up period, and the small size of the CD cohort. The absence of endoscopic and imaging assessment limited the ability to fully characterize disease activity, especially in CD patients. Additionally, the lack of routine fecal calprotectin testing limited our ability to assess inflammatory biomarkers. Given these limitations, early subjective improvements following initiation of a new therapy, including those influenced by cognitive bias or placebo effects, may have affected patients’ perception of self‐ PROs, underscoring the need for longer‐term randomized follow‐up studies that correlate subjective response measures with objective endoscopic, biochemical, or radiographic markers of disease.

## 5. Conclusions

This study demonstrated that improvements in QoL can occur as early as 2 weeks after the initiation of vedolizumab induction therapy. As predictors of reduced response were associated with the advanced disease, these findings highlight the importance of timely intervention. Prioritizing QoL improvement as a treatment goal aligns with patient‐reported experiences and should be firmly established as a key long‐term objective adjunctive to endoscopic and clinical response when assessing for long‐term sustained remission in the management of IBD.

## Author Contributions

Agata Michalak, Iwona Zawada, Anna Deskur, Maria Kopertowska‐Majchrzak, Renata Talar‐Wojnarowska, Danuta Domżał‐Magrowska, Piotr Eder, Kamila Stawczyk‐Eder, Katarzyna Waszak, Grażyna Rydzewska‐Wyszkowska, Katarzyna Maciejewska, Katarzyna Karłowicz, Anita Gąsiorowska, Aleksandra Kaczka, Łukasz Konecki, Michał Krogulecki, Aleksandra Filipiuk, Maria Kłopocka, Ariel Liebert, Marek Hartleb, Magdalena Gawron‐Kiszka, Elżbieta Poniewierka, Iga Gromny, Edyta Zagórowicz, Rafał Filip, and Halina Cichoż‐Lach: performance of the research, interpretation of the data, writing of the manuscript, revision of the manuscript, and final approval.

Eliza Majewska: interpretation of the data, writing of the manuscript, revision of the manuscript, and final approval.

## Funding

This study was sponsored by Takeda Pharma sp. z o.o., Warsaw, Poland. Writing support provided by Hanna Kurlanda‐Witek of Proper Medical Writing was funded by Takeda Pharma sp z o.o., Warsaw, Poland.

## Ethics Statement

This study was conducted in accordance with the Declaration of Helsinki, Good Clinical Practice (GCP), the International Society for Pharmacoepidemiology (ISPE) Guidelines for Good Pharmacoepidemiology Practice (GPP), and any applicable local regulations. The study protocol was approved by the Bioethics Committee of the Nicolaus Copernicus University in Toruń, Collegium Medicum in Bydgoszcz (Approval No. KB 36312020).

## Consent

All patients gave written consent to participate in the study.

## Conflicts of Interest

Agata Michalak received lecture fees and/or travel grants from AbbVie, Bayer, Biocodex, and Takeda. Iwona Zawada received lecture fees and/or travel grants from AbbVie, Takeda, Janssen, Recordati, Ferring, and Biocodex. Renata Talar‐Wojnarowska received lecture fees and/or travel grants from AbbVie, Astellas, Bristol Myers Squibb, Celltrion, Ferring, Janssen, Pfizer, Recordati, and Takeda. Piotr Eder received lecture fees or travel grants from AbbVie, Takeda, Pfizer, Ferring, Bristol Myers Squibb, Eli Lilly, Sanofi, SOBI, and ProMed. Kamila Stawczyk‐Eder received travel grants and lecture fees from Janssen, Pfizer, and Takeda. Katarzyna Waszak received payment for travel/accommodation/meeting expenses from Janssen‐Cilag, Pfizer, PRO.MED.CS, Samsung Bioepis, and Takeda. Grażyna Rydzewska‐Wyszkowska received speaker or consultant fees from AbbVie, AlfaSigma, AstraZeneca, Bayer, Biocodex, Bristol Mayers Squibb, Ferring Pharmaceuticals, Janssen, Lilly, PRO.MED.PL, Recordati, Sanprobi, SOBI, and Takeda. Katarzyna Maciejewska received lecture fees and/or travel grants from AbbVie, Takeda, Sandoz, Sobi, and Bristol Myers Squibb. Katarzyna Karłowicz received lecture fees, consultancy fees, or travel educational grants from Bristol Myers Squibb, AbbVie, Polpharma, Takeda, and Pfizer. Anita Gąsiorowska received lecture fees or travel grants from AbbVie, Ferring, Takeda, and ProMed. Aleksandra Kaczka received conference registration fee and/or travel grants from Takeda, AbbVie, and Sandoz. Michał Krogulecki received lecture fees and/or travel grants from AbbVie, Ferring, Janssen, Pfizer, Takeda, and Sobi. Aleksandra Filipiuk received payment for lectures from Janssen and Pfizer and travel/accommodation/meeting expenses from Takeda and Janssen. Maria Kłopocka received lecture fees or travel grants from Janssen, Takeda, Ferring, AbbVie, and Recordati. Ariel Liebert received payment for lectures from Janssen, Takeda, Egis, AbbVie, and Pharmabest and travel/accommodation/meeting expenses from Janssen, Takeda, Egis, and AbbVie. Magdalena Gawron‐Kiszka received lecture fees and/or travel grants from AbbVie, Eli Lilly, Ferring, Janssen, Pfizer, Recordati, and Takeda. Edyta Zagórowicz received lecture fees and/or travel grants from AbbVie, Eli Lilly, Janssen, Takeda, and Bristol Myers Squibb. Eliza Majewska is the permanent employee of Takeda Pharma sp z o.o., Warsaw, Poland. The other authors declare no conflicts of interest.

## Supporting Information

Additional supporting information can be found online in the Supporting Information section.

## Supporting information


**Supporting Information** Please refer to the Supporting Data for the following information: The STROBE checklist for observational studies. Supporting Table 1. Additional patient characteristics. Supporting Table 2. Psychoactive medication use during the study. Supporting Table 3. Full listing of values for IBDQ, FACIT‐Fatigue, and PROMIS questionnaires at Weeks 0, 2, 6, and 14, including subscales. Supporting Table 4. Changes in IBDQ, PROMIS, and FACIT scores relative to baseline based on clinical response to treatment. Supporting Table 5. Changes in IBDQ, PROMIS, and FACIT scores relative to baseline based on the use of psychoactive medication at any time during the study. Supporting Table 6. Full listing of laboratory parameters at baseline and at Week 14.

## Data Availability

The datasets generated and/or analyzed during the current study are available from the corresponding author upon reasonable request from researchers who provide a methodologically sound proposal. The data will be provided after their deidentification, in compliance with applicable privacy laws, data protection, and requirements for consent and anonymization.
