# Supplementary material for: Clinical Response and Improvement of the Quality of Life, Fatigue, Mood, and Sleep Disturbances During Vedolizumab Induction Therapy in Polish Patients With Moderate‐to‐Severe Ulcerative Colitis or Crohn’s Disease
Source: Can J Gastroenterol Hepatol. 2026 Jun 2;2026:9955621. doi: 10.1155/cjgh/9955621 (PMC13580841; doi:10.1155/cjgh/9955621)
Supplement: Supplementary file 1 [file CJGH-2026-9955621-s001.zip › 9955621_KUJAWIAK Study_MSS_TAG_DV-007110_Supplementary Data_revision2_clean_rev.docx]

**Supplementary Table 1**. Additional patient characteristics (FAS)

| **Parameter** | **Overall, N = 257** | **Patients with UC, n = 205** | **Patients with CD, n = 52** |
| --- | --- | --- | --- |
|  |  |  |  |
| **Disease** | 257 (100.0%) | 205 (79.8%) | 52 (20.2%) |
| **Duration of disease [years]** |  |  |  |
| n | 257 | 205 | 52 |
| Mean (SD) | 8.6 (6.6) | 8.1 (6.6) | 10.5 (6.2) |
| Median (min-max) | 7.0 (0.0 - 47.0) | 7.0 (0.0 - 47.0) | 8.5 (1.0 - 29.0) |
| Missing values | 0 | 0 | 0 |
| **Montreal classification; UC** |  |  |  |
| **Disease extent at the last colonoscopy** |  |  |  |
| E1 – ulcerative proctitis |  | 5 (2.4%) |  |
| E2 – left-sided UC |  | 98 (47.8%) |  |
| E3 – extensive UC |  | 102 (49.8%) |  |
| Missing data |  | 0 |  |
| **Disease severity** |  |  |  |
| S0 – clinical remission |  | 0 (0.0%) |  |
| S1 – mild UC |  | 8 (3.9%) |  |
| S2 – moderate UC |  | 150 (73.2%) |  |
| S3 – severe UC |  | 47 (22.9%) |  |
| Missing data |  | 0 |  |
| **Montreal classification; CD** |  |  |  |
| **Patient age at diagnosis** |  |  |  |
| A1 – below 16 years |  |  | 7 (13.5%) |
| A2 – between 17 and 40 years |  |  | 39 (75.0%) |
| A3 – over 40 years |  |  | 6 (11.5%) |
| Missing data |  |  | 0 |
| **Localization** |  |  |  |
| L1 – ileal |  |  | 9 (17.3%) |
| L2 – colonic |  |  | 11 (21.2%) |
| L3 – ileocolonic |  |  | 32 (61.5%) |
| L4 – isolated upper disease* |  |  | 1 (1.9%) |
| Missing data |  |  | 0 |
| **Disease behavior** |  |  |  |
| B1 – non-stricturing, non-penetrating |  |  | 19 (36.5%) |
| B2 – stricturing |  |  | 22 (42.3%) |
| B3 – penetrating |  |  | 11 (21.2%) |
| Missing data |  |  | 0 |
| P – perianal disease modifier |  |  | 5 (9.6%) |
| Missing data |  |  | 0 |
| **Occurrence of extraintestinal symptoms in the past** | 112 (43.6%) | 82 (40.0%) | 30 (57.7%) |
| **Occurrence of extraintestinal symptoms** | 81 (31.5%) | 53 (25.9%) | 28 (53.8%) |
| **Extraintestinal symptoms** |  |  |  |
| Arthralgia | 48 (18.7%) | 34 (16.6%) | 14 (26.9%) |
| Arthritis | 3 (1.2%) | 2 (1.0%) | 1 (1.9%) |
| Ankylosing spondylitis | 0 (0.0%) | 0 (0.0%) | 0 (0.0%) |
| Erythema nodosum | 3 (1.2%) | 2 (1.0%) | 1 (1.9%) |
| Pyoderma gangrenosum | 1 (0.4%) | 1 (0.5%) | 0 (0.0%) |
| Eye symptoms (iritis or scleritis) | 0 (0.0%) | 0 (0.0%) | 0 (0.0%) |
| Primary sclerosing cholangitis | 4 (1.6%) | 3 (1.5%) | 1 (1.9%) |
| Aphthous stomatitis | 7 (2.7%) | 3 (1.5%) | 4 (7.7%) |
| Nephrolithiasis | 4 (1.6%) | 1 (0.5%) | 3 (5.8%) |
| Cholelithiasis | 3 (1.2%) | 0 (0.0%) | 3 (5.8%) |
| Anemia | 42 (16.3%) | 26 (12.7%) | 16 (30.8%) |
| Missing data | 0 | 0 | 0 |
| **Occurrence of fistulas** | 4 (1.6%) | 0 (0.0%) | 4 (7.7%) |
| **Hospitalization due to worsening of symptoms^1^** |  |  |  |
| Yes | 164 (63.8%) | 129 (62.9%) | 35 (67.3%) |
| No | 93 (36.2%) | 76 (37.1%) | 17 (32.7%) |
| Missing data | 0 | 0 | 0 |
| **Number of hospitalizations** |  |  |  |
| 1 | 105 (40.9%) | 83 (40.5%) | 22 (42.3%) |
| 2 | 38 (14.8%) | 28 (13.7%) | 10 (19.2%) |
| More than 2 | 21 (8.2%) | 18 (8.8%) | 3 (5.8%) |
| Missing data | 0 | 0 | 0 |
| **Duration of hospitalization** |  |  |  |
| n | 164 | 129 | 35 |
| Mean (SD) | 10.1 (9.4) | 10.7 (10.2) | 7.8 (4.5) |
| Median (min-max) | 7.5 (0.0 - 59.0) | 8.0 (0.0 - 59.0) | 7.0 (1.0 - 17.0) |
| Q1 - Q3 | 4.0 - 13.0 | 4.0 - 14.0 | 4.5 - 11.0 |
| Missing values | 93 | 76 | 17 |

^1^ within the 12-month period preceding the study

Abbreviations: CD, Crohn’s disease; FAS, full analysis set; UC, ulcerative disease; *more than 1 localization was provided

**Supplementary Table 2.** Psychoactive medication use during the study (FAS)

| **Treatment subgroup** | **Visit 1 (week 0) N = 257** | **Visit 2 (week 2) n = 257** | **Visit 3 (week 6) n = 254** | **Visit 4 (week 14) n = 239** |
| --- | --- | --- | --- | --- |
| Any, n (%) | 24 (9.3%) | 20 (7.8%) | 22 (8.7%) | 23 (9.6%) |
| Antidepressants | 19 (7.4%) | 18 (7%) | 20 (7.9%) | 21 (8.8%) |
| Antiepileptics | 2 (0.8%) | 2 (0.8%) | 3 (1.2%) | 3 (1.3%) |
| Antipsychotics | 3 (1.2%) | 3 (1.2%) | 3 (1.2%) | 4 (1.7%) |
| Anxiolytics | 3 (1.2%) | 3 (1.2%) | 3 (1.2%) | 2 (0.8%) |
| Drugs used in addictive disorders | 1 (0.4%) | 0 (0%) | 0 (0%) | 0 (0%) |
| Hypnotics and sedatives | 2 (0.8%) | 1 (0.4%) | 1 (0.4%) | 1 (0.4%) |
| Percentage calculated on basis of all patients that participated in the visit  FAS, full analysis set | | | | |

**Supplementary Table 3.** Full listing of values for IBDQ, FACIT-Fatigue, and PROMIS questionnaires at weeks 0, 2, 6, and 14, including subscales.

**A** IBDQ scores and subscales at baseline and at week 2, 6, and 14 (FAS)

| **Parameter** | **Overall, N = 257** | **Patients with UC, n = 205** | **Patients with CD, n = 52** |
| --- | --- | --- | --- |
| **Visit 1 (week 0)** |  |  |  |
| **Bowel symptoms** |  |  |  |
| n | 257 | 205 | 52 |
| Mean (SD) | 42.2 (11.2) | 41.6 (11.3) | 44.5 (10.9) |
| Median (min-max) | 41.0 (17.0 - 70.0) | 41.0 (17.0 - 70.0) | 44.0 (21.0 - 67.0) |
| Missing values | 0 | 0 | 0 |
| **Systemic symptoms** |  |  |  |
| n | 257 | 205 | 52 |
| Mean (SD) | 17.9 (6.5) | 17.9 (6.5) | 17.8 (6.6) |
| Median (min-max) | 17.0 (6.0 - 34.0) | 17.0 (6.0 - 34.0) | 17.5 (6.0 - 31.0) |
| Missing values | 0 | 0 | 0 |
| **Emotional function** |  |  |  |
| n | 257 | 205 | 52 |
| Mean (SD) | 48.4 (14.1) | 48.1 (14.2) | 49.6 (13.9) |
| Median (min-max) | 49.0 (14.0 - 81.0) | 48.0 (14.0 - 81.0) | 49.5 (18.0 - 76.0) |
| Missing values | 0 | 0 | 0 |
| **Social function** |  |  |  |
| n | 257 | 205 | 52 |
| Mean (SD) | 19.5 (7.9) | 19.1 (8.0) | 21.1 (7.4) |
| Median (min-max) | 20.0 (5.0 - 35.0) | 19.0 (5.0 - 35.0) | 22.0 (6.0 - 33.0) |
| Missing values | 0 | 0 | 0 |
| **IBDQ total score** |  |  |  |
| n | 257 | 205 | 52 |
| Mean (SD) | 128.0 (34.8) | 126.7 (34.9) | 133.0 (33.9) |
| Median (min-max) | 127.0 (50.0 - 215.0) | 125.0 (50.0 - 215.0) | 132.0 (61.0 - 199.0) |
| Missing values | 0 | 0 | 0 |
| **Visit 2 (week 2)** |  |  |  |
| **Bowel symptoms** |  |  |  |
| n | 256 | 204 | 52 |
| Mean (SD) | 49.3 (11.6) | 48.8 (12.2) | 50.2 (11.2) |
| Median (min-max) | 50.5 (19.0 - 70.0) | 50.0 (19.0 - 70.0) | 53.0 (21.0 - 68.0) |
| Missing values | 1 | 1 | 0 |
| **Systemic symptoms** |  |  |  |
| n | 256 | 204 | 52 |
| Mean (SD) | 20.6 (6.2) | 20.6 (6.1) | 20.5 (6.9) |
| Median (min-max) | 21.0 (6.0 - 34.0) | 20.0 (6.0 - 34.0) | 22.0 (6.0 - 32.0) |
| Missing values | 1 | 1 | 0 |
| **Emotional function** |  |  |  |
| n | 256 | 204 | 52 |
| Mean (SD) | 54.9 (14.7) | 54.8 (14.6) | 55.7 (15.1) |
| Median (min-max) | 56.0 (17.0 - 81.0) | 55.0 (17.0 - 81.0) | 59.5 (20.0 - 79.0) |
| Missing values | 1 | 1 | 0 |
| **Social function** |  |  |  |
| n | 256 | 204 | 52 |
| Mean (SD) | 22.6 (8.0) | 22.2 (8.3) | 24.1 (6.6) |
| Median (min-max) | 24.0 (4.0 - 35.0) | 23.0 (4.0 - 35.0) | 25.0 (8.0 - 35.0) |
| Missing values | 1 | 1 | 0 |
| **IBDQ total score** |  |  |  |
| n | 256 | 204 | 52 |
| Mean (SD) | 147.4 (36.0) | 146.6 (36.3) | 150.5 (35.1) |
| Median (min-max) | 147.0 (59.0 - 219.0) | 146.0 (62.0 - 219.0) | 159.0 (59.0 - 206.0) |
| Missing values | 1 | 1 | 0 |
| **Visit 3 (week 6)** |  |  |  |
| **Bowel symptoms** |  |  |  |
| n | 257 | 205 | 52 |
| Mean (SD) | 52.4 (12.0) | 52.7 (11.9) | 51.2 (12.5) |
| Median (min-max) | 55.0 (21.0 - 70.0) | 55.0 (21.0 - 70.0) | 53.5 (21.0 - 69.0) |
| Missing values | 0 | 0 | 0 |
| **Systemic symptoms** |  |  |  |
| n | 257 | 205 | 52 |
| Mean (SD) | 22.1 (6.7) | 22.1 (6.4) | 21.9 (7.7) |
| Median (min-max) | 23.0 (6.0 - 34.0) | 23.0 (7.0 - 34.0) | 24.0 (6.0 - 33.0) |
| Missing values | 0 | 0 | 0 |
| **Emotional function** |  |  |  |
| n | 257 | 205 | 52 |
| Mean (SD) | 57.2 (15.8) | 57.0 (15.2) | 57.7 (18.0) |
| Median (min-max) | 60.0 (13.0 - 83.0) | 59.0 (13.0 - 83.0) | 62.5 (20.0 - 82.0) |
| Missing values | 0 | 0 | 0 |
| **Social function** |  |  |  |
| n | 257 | 205 | 52 |
| Mean (SD) | 25.0 (8.2) | 24.8 (8.3) | 25.5 (7.8) |
| Median (min-max) | 27.0 (5.0 - 35.0) | 26.0 (5.0 - 35.0) | 28.0 (7.0 - 35.0) |
| Missing values | 0 | 0 | 0 |
| **IBDQ total score** |  |  |  |
| n | 257 | 205 | 52 |
| Mean (SD) | 156.6 (38.5) | 156.7 (37.3) | 156.3 (43.2) |
| Median (min-max) | 162.0 (50.0 - 218.0) | 162.0 (50.0 - 218.0) | 165.0 (59.0 - 215.0) |
| Missing values | 0 | 0 | 0 |
| **Visit 4 (week 14)** |  |  |  |
| **Bowel symptoms** |  |  |  |
| n | 257 | 205 | 52 |
| Mean (SD) | 52.8 (13.5) | 53.3 (13.8) | 50.9 (12.0) |
| Median (min-max) | 56.0 (0.0 - 70.0) | 58.0 (0.0 - 70.0) | 54.0 (21.0 - 70.0) |
| Missing values | 0 | 0 | 0 |
| **Systemic symptoms** |  |  |  |
| n | 255 | 203 | 52 |
| Mean (SD) | 23.2 (6.6) | 23.4 (6.4) | 22.2 (7.4) |
| Median (min-max) | 24.0 (6.0 - 35.0) | 25.0 (7.0 - 34.0) | 24.0 (6.0 - 35.0) |
| Missing values | 2 | 2 | 0 |
| **Emotional function** |  |  |  |
| n | 255 | 203 | 52 |
| Mean (SD) | 58.93 (16.6) | 58.7 (16.4) | 56.8 (17.5) |
| Median (min-max) | 62.0 (13.0 - 84.0) | 62.0 (13.0 - 83.0) | 61.0 (20.0 - 84.0) |
| Missing values | 2 | 2 | 0 |
| **Social function** |  |  |  |
| n | 255 | 203 | 52 |
| Mean (SD) | 26.0 (8.4) | 26.0 (8.5) | 25.8 (7.8) |
| Median (min-max) | 29.0 (5.0 - 35.0) | 29.0 (5.0 - 35.0) | 28.0 (7.0 - 35.0) |
| Missing values | 2 | 2 | 0 |
| **IBDQ total score** |  |  |  |
| n | 255 | 203 | 52 |
| Mean (SD) | 160.7 (40.7) | 162.0 (40.4) | 155.8 (41.7) |
| Median (min-max) | 169.0 (50.0 - 223.0) | 170.0 (50.0 - 222.0) | 163.5 (59.0 - 223.0) |
| Missing values | 2 | 2 | 0 |

**B** FACIT-Fatigue prorated scores at baseline and at week 2, 6, and 14 (FAS)

| **Parameter**  **Visit 1 (week 0)** | **Patients with UC, n = 205** | **Patients with CD, n = 52** |
| --- | --- | --- |
| **Prorated score** |  |  |
| n | 205 | 52 |
| Mean (SD) | 28.3 (10.5) | 27.0 (11.0) |
| Median (min-max) | 28.0 (4.0 - 51.0) | 26.0 (6.0 - 49.0) |
| Missing values | 0 | 0 |
| **Visit 2 (week 2)** |  |  |
| **Prorated score** |  |  |
| n | 203 | 52 |
| Mean (SD) | 31.3 (10.2) | 30.2 (11.1) |
| Median (min-max) | 32.0 (7.0 - 52.0) | 32.0 (8.0 - 49.0) |
| Missing values | 2 | 0 |
| **Visit 3 (week 6)** |  |  |
| **Prorated score** |  |  |
| n | 205 | 50 |
| Mean (SD) | 32.6 (10.5) | 32.5 (12.9) |
| Median (min-max) | 34.0 (3.0 - 51.0) | 33.5 (6.0 - 52.0) |
| Missing values | 0 | 2 |
| **Visit 4 (week 14)** |  |  |
| **Prorated score** |  |  |
| n | 202 | 52 |
| Mean (SD) | 34.8 (11.2) | 32.7 (12.8) |
| Median (min-max) | 37.0 (3.0 - 52.0) | 34.5 (6.0 - 52.0) |
| Missing values | 3 | 0 |

| **C** PROMIS prorated T-scores at baseline and at week 2, 6, and 14 (FAS) | | |
| --- | --- | --- |
| **Visit 1 (week 0)** | **Patients with UC, n = 205** | **Patients with CD, n = 52** |
| **Sleep disturbance** |  |  |
| n | 205 | 52 |
| Mean (SD) | 53.8 (8.6) | 54.4 (9.4) |
| Median (min-max) | 54.3 (28.9 - 73.0) | 53.8 (28.9 - 73.0) |
| Missing values | 0 | 0 |
| **Depression** |  |  |
| n | 205 | 52 |
| Mean (SD) | 56.7 (7.8) | 56.8 (8.7) |
| Median (min-max) | 56.2 (37.1 - 77.9) | 55.8 (37.1 - 76.2) |
| Missing values | 0 | 0 |
| **Visit 2 (week 2)** |  |  |
| **Sleep disturbance** |  |  |
| n | 204 | 52 |
| Mean (SD) | 52.5 (8.9) | 51.9 (10.4) |
| Median (min-max) | 53.3 (28.9 - 76.5) | 53.2 (28.9 - 73.0) |
| Missing values | 1 | 0 |
| **Depression** |  |  |
| n | 204 | 52 |
| Mean (SD) | 54.5 (8.5) | 54.8 (8.9) |
| Median (min-max) | 54.3 (37.1 - 81.1) | 54.8 (37.1 - 73.6) |
| Missing values | 1 | 0 |
| **Visit 3 (week 6)** |  |  |
| **Sleep disturbance** |  |  |
| n | 205 | 52 |
| Mean (SD) | 51.7 (8.9) | 50.6 (10.6) |
| Median (min-max) | 52.2 (28.9 - 76.5) | 51.2 (28.9 - 76.5) |
| Q1 - Q3 | 45.5 - 58.3 | 41.4 - 59.4 |
| Missing values | 0 | 0 |
| **Depression** |  |  |
| n | 205 | 51 |
| Mean (SD) | 53.4 (8.7) | 53.8 (9.9) |
| Median (min-max) | 53.4 (37.1 - 81.1) | 52.3 (37.1 - 72.5) |
|  | 0 | 1 |
| **Visit 4 (week 14)** |  |  |
| **Sleep disturbance** |  |  |
| n | 203 | 52 |
| Mean (SD) | 49.8 (8.9) | 50.4 (9.6) |
| Median (min-max) | 50.1 (28.9 - 76.5) | 50.1 (28.9 - 73.0) |
| Missing values | 2 | 0 |
| **Depression** |  |  |
| n | 203 | 52 |
| Mean (SD) | 53.1 (9.4) | 54.0 (10.0) |
| Median (min-max) | 52.3 (37.1 - 81.1) | 52.8 (37.1 - 72.5) |
| Missing values | 2 | 0 |

For early termination patients, missing data were imputed using the LOCF method, that is the last non-missing value used to impute missing values on subsequent visits.

Abbreviations: CD, Crohn’s disease; FACIT-F, Functional Assessment of Chronic Illness Therapy-Fatigue; FAS, full analysis set; IBDQ, Inflammatory Bowel Disease Questionnaire; SD, standard deviation; UC, ulcerative disease

**Supplementary Table 4.** Changes in IBDQ, PROMIS and FACIT scores relative to baseline based on clinical response to treatment (FAS). Response at week 14 is defined as a decrease of at least 3 points from baseline Mayo score for UC; CDAI scale by 70 points and 25% for CD patients.

| **Parameter** | **Patients with UC, N = 205** |  |  | **p-value** | **Patients with CD, N = 52** |  |  | **p-value** |
| --- | --- | --- | --- | --- | --- | --- | --- | --- |
|  | **Overall, N = 205** | **Responder, N = 168** | **Non-responder, N = 37** | Responder vs Non-responder | **Overall, N = 52** | **Responder, N = 43** | **Non-responder, N = 9** | Responder vs Non-responder |
| **Difference from baseline vs Week 14 in IBDQ total score** |  |  |  | <0.001 (t-test) |  |  |  | 0.011 (t-test) |
| N | 203 | 167 | 36 |  | 52 | 43 | 9 |  |
| mean (SD) | 35.1 (41.5) | 42.3 (39.0) | 1.8 (36.4) |  | 22.8 (34.6) | 28.1 (33.9) | -2.6 (26.9) |  |
| median (min-max) | 29.0 (-80.0 - 172.0) | 40.0 (-51.0 - 172.0) | 8.0 (-80.0 - 111.0) |  | 20.0 (-48.0 - 144.0) | 24.0 (-48.0 - 144.0) | -3.0 (-44.0 - 50.0) |  |
| 95% CI for mean | 35.3 (30.3; 40.3) [boot] | 39.8 (34.6; 45.0) [boot] | 7.7 (-4.7; 20.1) [t] |  | 26.9 (18.0; 36.7) [boot] | 28.1 (17.6; 38.5) [t] | 14.8 (-30.3; 59.8) [t] |  |
| Test | Wilcoxon signed rank test with continuity correction | Wilcoxon signed rank test with continuity correction | Paired t-test |  | Wilcoxon signed rank test with continuity correction | Paired t-test | Paired t-test |  |
| p-value | <0.001 | <0.001 | 0.215 |  | <0.001 | <0.001 | 1.000 |  |
| **Difference from baseline vs Week 14 in FACIT-Fatigue prorated score** |  |  |  | <0.001 (Wilcoxon) |  |  |  | 0.589 (t-test) |
| N | 202 | 166 | 36 |  | 52 | 43 | 9 |  |
| mean (SD) | 6.4 (11.5) | 7.8 (11.0) | -0.2 (11.6) |  | 5.7 (9.6) | 6.0 (9.6) | 4.0 (9.9) |  |
| median (min-max) | 5.0 (-27.0 - 39.0) | 6.0 (-21.0 - 39.0) | 0.5 (-27.0 - 22.0) |  | 5.0 (-11.0 - 35.0) | 6.0 (-11.0 - 35.0) | 2.0 (-10.0 - 23.0) |  |
| 95% CI for mean | 6.4 (4.8; 8.0) [boot] | 7.8 (6.1; 9.5) [boot] | -0.2 (-4.2; 3.7) [t] |  | 5.7 (3.0; 8.3) [t] | 6.0 (3.1; 8.9) [t] | 4.0 (-3.6; 11.6) [t] |  |
| Test | Wilcoxon signed rank test with continuity correction | Wilcoxon signed rank test with continuity correction | Paired t-test |  | Paired t-test | Paired t-test | Paired t-test |  |
| p-value | <0.001 | <0.001 | 1.000 |  | <0.001 | <0.001 | 0.775 |  |
| **Difference from baseline vs Week 14 in PROMIS Sleep disturbance prorated T-score** |  |  |  | 0.003 (Wilcoxon) |  |  |  | 0.105 (Wilcoxon) |
| N | 203 | 167 | 36 |  | 52 | 43 | 9 |  |
| mean (SD) | -4.0 (9.1) | -4.8 (9.2) | -0.5 (7.6) |  | -3.9 (7.7) | -4.7 (7.9) | -0.2 (5.0) |  |
| median (min-max) | -4.1 (-40.1 - 31.5) | -4.6 (-40.1 - 31.5) | -0.5 (-22.0 - 23.2) |  | -3.2 (-31.6 - 10.9) | -3.4 (-31.6 - 10.9) | -1.0 (-7.2 - 8.3) |  |
| 95% CI for mean | -4.0 (-5.3; -2.8) [boot] | -4.8 (-6.1; -3.4) [boot] | -0.5 (-3.0; 2.1) [t] |  | -3.9 (-6.1; -1.8) [t] | -4.7 (-7.2; -2.4) [boot] | -0.2 (-4.1; 3.7) [t] |  |
| Test | Wilcoxon signed rank test with continuity correction | Wilcoxon signed rank test with continuity correction | Paired t-test |  | Paired t-test | Wilcoxon signed rank test with continuity correction | Paired t-test |  |
| p-value | <0.001 | <0.001 | 1.000 |  | 0.001 | <0.001 | 1.000 |  |
| **Difference from baseline vs Week 14 in prorated T-score for depression** |  |  |  | <0.001 (Wilcoxon) |  |  |  | 0.191 (Wilcoxon) |
| N | 203 | 167 | 36 |  | 52 | 43 | 9 |  |
| mean (SD) | -3.5 (8.3) | -4.6 (8.1) | 1.3 (7.3) |  | -2.8 (8.2) | -3.4 (8.7) | 0.2 (5.2) |  |
| median (min-max) | -2.0 (-34.3 - 20.0) | -3.0 (-34.3 - 14.6) | 0.0 (-12.7 - 20.0) |  | -2.3 (-32.9 - 14.6) | -2.9 (-32.9 - 14.6) | 0.0 (-8.0 - 8.9) |  |
| 95% CI for mean | -3.5 (-4.7; -2.4) [boot] | -4.6 (-5.8; -3.4) [boot] | 1.3 (-1.1; 3.8) [t] |  | -2.8 (-5.0; -0.6) [boot] | -3.4 (-6.1; -0.9) [boot] | 0.2 (-3.8; 4.1) [t] |  |
| Test | Wilcoxon signed rank test with continuity correction | Wilcoxon signed rank test with continuity correction | Paired t-test |  | Wilcoxon signed rank test with continuity correction | Wilcoxon signed rank test with continuity correction | Paired t-test |  |
| p-value | <0.001 | <0.001 | 0.568 |  | 0.024 | 0.013 | 1.000 |  |

For early termination patients, missing data were imputed using the LOCF method, that is the last non-missing value used to impute missing values on subsequent visits.

Abbreviations: CD, Crohn’s disease; FAS, full analysis set; SD, standard deviation; UC, ulcerative disease

| **Supplementary Table 5.** Changes in IBDQ, PROMIS, and FACIT scores relative to baseline based on use of psychoactive medication at any time during the study (FAS). **Parameter** \| **All patients, N = 257** \| **Psychoactive drugs, N = 30** \| **No psychoactive drugs, N = 227** \| **p-value**; \| \| \| \| Psychoactive Yes vs No; **Difference from baseline vs Week 14 in IBDQ total score** \| \| \| \| 0.138 (t-test); N \| 255 \| 29 \| 226 \|; mean (SD) \| 32.6 (40.4) \| 42.3 (36.0) \| 31.3 (40.8) \|; median (min-max) \| 27.0 (-80.0 - 172.0) \| 38.0 (-7.0 - 130.0) \| 26.0 (-80.0 - 172.0) \|; 95% CI for mean \| 35.3 (30.3; 40.3) [boot] \| 43.9 (30.1; 57.7) [t] \| 34.2 (29.0; 39.6) [boot] \|; Test \| Wilcoxon signed rank test with continuity correction \| Paired t-test \| Wilcoxon signed rank test with continuity correction \|; p-value \| <0.001 \| <0.001 \| <0.001 \|; **Difference from baseline vs Week 14 in prorated score** \| \| \| \| 0.146 (Wilcoxon); N \| 254 \| 29 \| 225 \|; mean (SD) \| 6.2 (11.1) \| 9.1 (11.5) \| 5.8 (11.0) \|; median (min-max) \| 5.0 (-27.0 - 39.0) \| 9.0 (-15.0 - 32.0) \| 5.0 (-27.0 - 39.0) \|; 95% CI for mean \| 6.2 (4.8; 7.6) [boot] \| 9.1 (4.8; 13.5) [t] \| 5.8 (4.4; 7.3) [boot] \|; Test \| Wilcoxon signed rank test with continuity correction \| Paired t-test \| Wilcoxon signed rank test with continuity correction \|; p-value \| <0.001 \| <0.001 \| <0.001 \|; **Difference from baseline vs Week 14 in prorated T-score for sleep disturbances** \| \| \| \| 0.536 (Wilcoxon); N \| 255 \| 29 \| 226 \|; mean (SD) \| -4.0 (8.8) \| -3.0 (5.6) \| -4.1 (9.1) \|; median (min-max) \| -4.1 (-40.1 - 31.5) \| -3.1 (-13.7 - 8.3) \| -4.1 (-40.1 - 31.5) \|; 95% CI for mean \| -4.0 (-5.0; -2.9) [boot] \| -3.0 (-5.1; -0.8) [t] \| -4.0 (-5.4; -2.6) [boot] \|; Test \| Wilcoxon signed rank test with continuity correction \| Paired t-test \| Wilcoxon signed rank test with continuity correction \|; p-value \| <0.001 \| 0.025 \| <0.001 \|; **Difference from baseline vs Week 14 in prorated T-score for depression** \| \| \| \| 0.166 (Wilcoxon); N \| 255 \| 29 \| 226 \|; mean (SD) \| -3.4 (8.3) \| -5.4 (9.4) \| -3.1 (8.1) \|; median (min-max) \| -2.0 (-34.3 - 20.0) \| -5.6 (-26.7 - 11.4) \| -2.0 (-34.3 - 20.0) \|; 95% CI for mean \| -3.4 (-4.4; -2.4) [boot] \| -5.4 (-9.0; -1.8) [t] \| -3.1 (-4.2; -2.1) [boot] \|; Test \| Wilcoxon signed rank test with continuity correction \| Paired t-test \| Wilcoxon signed rank test with continuity correction \|; p-value \| <0.001 \| 0.014 \| <0.001 \| For early termination patients, missing data were imputed using the LOCF method, that is the last non-missing value used to impute missing values on subsequent visits. Abbreviations: FAS, full analysis set; SD, standard deviation **Supplementary Table 6.** Full listing of laboratory parameters at baseline and at week 14 (FAS). | | | |
| --- | --- | --- | --- |
| **Visit 1 (week 0)** | **Patients with UC, n = 205** | **Patients with CD, n = 52** |  |
| **CRP [mg/l]** |  |  |  |
| N | 204 | 52 |  |
| Mean (SD) | 10.8 (19.9) | 15.9 (22.3) |  |
| Median (min-max) | 4.5 (0.1 - 207.0) | 9.0 (0.2 - 114.0) |  |
| Missing values | 1 | 0 |  |
| **Hemoglobin [g/dl]** |  |  |  |
| N | 205 | 52 |  |
| Mean (SD) | 13.1 (2.0) | 12.7 (1.7) |  |
| Median (min-max) | 13.3 (7.4 - 17.8) | 12.8 (8.5 - 15.8) |  |
| Missing values | 0 | 0 |  |
| **Hematocrit [%]** |  |  |  |
| N | 205 | 52 |  |
| Mean (SD) | 39.7 (5.2) | 38.5 (4.7) |  |
| Median (min-max) | 39.9 (24.2 - 51.3) | 38.1 (28.4 - 48.4) |  |
| Missing values | 0 | 0 |  |
| **Red blood cells [10^12/l]** |  |  |  |
| N | 204 | 52 |  |
| Mean (SD) | 4.5 (0.6) | 4.4 (0.6) |  |
| Median (min-max) | 4.5 (2.9 - 6.1) | 4.6 (2.9 - 5.8) |  |
| Missing values | 1 | 0 |  |
| **MCV [fl]** |  |  |  |
| n | 205 | 52 |  |
| Mean (SD) | 88.6 (6.8) | 88.4 (8.0) |  |
| Median (min-max) | 89.0 (67.4 - 104.1) | 88.3 (65.5 - 107.2) |  |
| Missing values | 0 | 0 |  |
| **MCH [pg]** |  |  |  |
| n | 204 | 52 |  |
| Mean (SD) | 29.2 (3.0) | 29.2 (3.3) |  |
| Median (min-max) | 29.5 (19.0 - 35.5) | 29.8 (19.8 - 35.1) |  |
| Missing values | 1 | 0 |  |
| **Platelet count [10^9/l]** |  |  |  |
| n | 205 | 52 |  |
| Mean (SD) | 335.4 (105.5) | 344.6 (131.2) |  |
| Median (min-max) | 317.0 (57.0 - 800.0) | 335.0 (139.0 - 977.0) |  |
| Missing values | 0 | 0 |  |
| **Visit 4 (week 14)** |  |  |  |
| **CRP [mg/l]** |  |  |  |
| n | 203 | 51 |  |
| Mean (SD) | 6.3 (9.3) | 18.8 (29.1) |  |
| Median (min-max) | 3.1 (0.1 - 72.3) | 8.5 (0.5 - 118.1) |  |
| Missing values | 2 | 1 |  |
| **Hemoglobin [g/dl]** |  |  |  |
| n | 204 | 51 |  |
| Mean (SD) | 13.5 (2.0) | 12.8 (1.6) |  |
| Median (min-max) | 13.8 (6.0 - 18.7) | 12.9 (9.3 - 15.9) |  |
| Missing values | 1 | 1 |  |
| **Hematocrit [%]** |  |  |  |
| n | 204 | 51 |  |
| Mean (SD) | 40.7 (4.6) | 38.6 (4.0) |  |
| Median (min-max) | 41.0 (21.4 - 52.7) | 39.0 (31.0 - 46.9) |  |
| Missing values | 1 | 1 |  |
| **Red blood cells [10^12/l]** |  |  |  |
| n | 204 | 51 |  |
| Mean (SD) | 4.7 (0.6) | 4.4 (0.5) |  |
| Median (min-max) | 4.6 (2.9 - 6.4) | 4.5 (3.1 - 5.8) |  |
| Missing values | 1 | 1 |  |
| **MCV [fl]** |  |  |  |
| n | 204 | 51 |  |
| Mean (SD) | 87.8 (6.8) | 88.7 (7.7) |  |
| Median (min-max) | 88.6 (68.5 - 105.2) | 88.2 (65.7 - 109.1) |  |
| Missing values | 1 | 1 |  |
| **MCH [pg]** |  |  |  |
| n | 204 | 51 |  |
| Mean (SD) | 29.2 (3.0) | 29.4 (3.1) |  |
| Median (min-max) | 29.5 (19.7 - 37.7) | 29.6 (19.1 - 34.7) |  |
| Missing values | 1 | 1 |  |
| **Platelet count [10^9/l]** |  |  |  |
| n | 204 | 51 |  |
| Mean (SD) | 315.9 (93.2) | 322.5 (102.3) |  |
| Median (min-max) | 304.5 (133.0 - 705.0) | 310.0 (141.0 - 686.0) |  |
| Missing values | 1 | 1 |  |

For early termination patients, missing data were imputed using the LOCF method, that is the last non-missing value used to impute missing values on subsequent visits.

Abbreviations: CD, Crohn’s disease; CRP, C-reactive protein; FAS, full analysis set; MCH, mean corpuscular hemoglobin; MCV, mean corpuscular volume; UC, ulcerative disease
